
JOURNAL INFORMATION
==============================
NLM Title Abbreviation: Biospektrum (Heidelb)
Journal ID: Biospektrum (Heidelb)
NLM Journal ID: 9615685

Biospektrum

ISSN: 0947-0867
EISSN: 1868-6249

ARTICLE INFORMATION
==============================
PMCID: PMC10230460
PMID: 37275942
DOI: 10.1007/s12268-023-1924-9
Article ID: 1924
Article version: 1
Subjects: Editorial

Ein Herz für Viren!

Mettenleiter Thomas C. thomas.mettenleiter@fli.de

grid.417834.d Friedrich-Loeffler-Institut, Bundesforschungsinstitut für Tiergesundheit, D-17493 Greifswald-Insel Riems, Greifswald, Deutschland
Electronic publication date: 2023 May 31
Print publication date: 2023
Volume: 29
Issue: 3
First page: 235
Last page: 235
Copyright: © Der Autor 2023
License: Dieser Artikel wird unter der Creative Commons Namensnennung 4.0 International Lizenz veröffentlicht, welche die Nutzung, Vervielfältigung, Bearbeitung, Verbreitung und Wiedergabe in jeglichem Medium und Format erlaubt, sofern Sie den/die ursprünglichen Autor(en) und die Quelle ordnungsgemäß nennen, einen Link zur Creative Commons Lizenz beifügen und angeben, ob Änderungen vorgenommen wurden. Die in diesem Artikel enthaltenen Bilder und sonstiges Drittmaterial unterliegen ebenfalls der genannten Creative Commons Lizenz, sofern sich aus der Abbildungslegende nichts anderes ergibt. Sofern das betreffende Material nicht unter der genannten Creative Commons Lizenz steht und die betreffende Handlung nicht nach gesetzlichen Vorschriften erlaubt ist, ist für die oben aufgeführten Weiterverwendungen des Materials die Einwilligung des jeweiligen Rechteinhabers einzuholen. Weitere Details zur Lizenz entnehmen Sie bitte der Lizenzinformation auf http://creativecommons.org/licenses/by/4.0/deed.de.
License URL: https://creativecommons.org/licenses/by/4.0/

issue-copyright-statement: © Springer-Verlag GmbH Deutschland, ein Teil von Springer Nature 2023

==============================

■ Viren haben ein Imageproblem. Was sie anrichten können, mussten wir alle in den letzten Jahren der COVID-19-Pandemie schmerzlich erleben. Diese infektiösen Nanopartikel mit einem Schnipsel genetischen Materials haben uns eine globale Krise beschert und nicht nur direkt, sondern auch indirekt großen Schaden angerichtet. Viren sind also „Schädlinge“ wie andere Parasiten auch? Und unsere Aufgabe besteht in ihrer Bekämpfung, am besten bis zur globalen Eradikation, wie es bei den Pocken und der Rinderpest bereits gelungen ist? Viren sind die Erreger von Krankheiten bei Pflanze, Tier und Mensch und hier sind Prävention (z. B. Impfung) und Therapie als Teil der Bekämpfung angesagt. Aber Viren (oder virusähnliche Elemente) sind auch Treiber der Evolution, ja sie stehen am Beginn der Evolution. Ohne sie gäbe es uns nicht. Viren stellen die zahlreichste Lebensform auf diesem Planeten dar, sie repräsentieren die größte genetische Vielfalt. Es gibt mehr Viren auf der Erde als Sterne am Himmel, 10-mal mehr als Bakterien [1]. Viren gehören zu unserem Lebensraum, sind ständig um uns. Ist das nicht eine apokalyptische Vorstellung? Nein, wenn wir Viren weniger aus medizinischer, sondern mehr aus Sicht der Biologie betrachten, sind sie faszinierende Repräsentanten irdischen Lebens mit einem ungeheuren Einfluss auf alles, was sich auf diesem Planeten tut. Bakterienviren zum Beispiel, sogenannte Bakteriophagen, sind essenziell für den Stoffumsatz in den Weltmeeren, sie begrenzen das Wachstum von Bakterien inkl. Blaualgen und wirken damit ökologischen Katastrophen entgegen. Damit sind sie spannende Forschungsobjekte, um den Kreislauf des Lebens auf diesem Planeten im Grundsatz zu verstehen. Daher plädiere ich für eine vorurteilsfreie Sicht auf diese Nanowelt von Lebensformen, die wir bisher nur in Ansätzen kennen.

Im Gegensatz zu Viren haben Virolog:innen kein Imageproblem. Im Gegenteil: Es gibt derzeit wohl gefühlt ca. 80 Millionen Virolog:innen in Deutschland. Ungefähr so viele wie Fußball-Bundestrainer:innen. Kaum eine Talkshow in den letzten drei Jahren kam ohne mindestens eine Person entsprechender, manchmal selbsterklärter Expertise aus. Die COVID-19-Pandemie wurde jetzt für Deutschland „offiziell“ als beendet erklärt. Wir befinden uns also wieder in einer interpandemischen Phase. Diese Bezeichnung ist wohl gewählt, denn aus der Geschichte können wir lernen, dass nach der Pandemie immer auch vor der nächsten Pandemie ist. Was auch immer wir betrachten, ob die Pest im Mittelalter (in diesem Fall waren Bakterien die Erreger), die verschiedenen Episoden der Pocken, die großen Grippepandemien des letzten Jahrhunderts, SARS oder mPox.

Es wird derzeit oft postuliert, dass die Menschheit nun in eine pandemische Ära eintrete. Dies basiert auf einfachen Tatsachen: Die menschliche Population hat im letzten November acht Milliarden erreicht, die ernährt werden wollen und auch nach tierischem Protein verlangen. So werden immer mehr Nutztiere gehalten, besonders die weltweite Geflügelpopulation steigt exponentiell. Mehr Nutztiere brauchen mehr Futter, was wiederum die Erweiterung von Anbauflächen auf Kosten von bisher unberührten Ökosystemen bzw. Umnutzungen von Landflächen nach sich zieht. Dies beeinflusst nicht nur die Biodiversität, sondern auch den Klimawandel. Mehr Menschen und mehr Nutztiere gibt aber auch mehr Gelegenheit zur Interaktion. Für zoonotische Erreger ein Eldorado: Acht Milliarden potenzielle Wirte, die hoch mobil sind und sich bevorzugt in urbanen Räumen zusammenballen. Und die menschliche Population wächst weiter.

Stehen wir also vor einer pandemischen Apokalypse? Pandemien sind Teil der Menschheitsgeschichte und werden es wohl auch bleiben. Aber COVID-19 hat auch deutlich gemacht, dass wir nicht nur passive Opfer sind. Die rasante Entwicklung der neuartigen mRNA-Vakzine, die auf jahrzehntelangen Arbeiten zur Entwicklung von Nukleinsäure-Impfstoffen basiert, zeigt die Bedeutung von langfristigen Forschungsansätzen, die sich nicht in „Grundlagenforschung“ versus „Angewandte Forschung“ differenzieren lassen. Notwendig ist exzellente Forschung. Hierfür werden entsprechende Infrastrukturen gebraucht, in der meist kostenintensiven Realisierung UND in der nachhaltigen Finanzierung. Notwendig ist aber auch eine adäquate Ausstattung der Bildungsbereiche, die die nächsten Generationen von Forschenden für diese Felder begeistern. Notwendig ist auch die Abkehr vom Denken und Handeln in Silos zugunsten eines „One Health“-Ansatzes, der in der interdisziplinären und transsektoralen Zusammenarbeit von Human-, Tier- und Umweltwissenschaften neue Forschungspotenziale jenseits anthropozentrischer Fokussierung erschließt und so die Treiber potenziell riskanter Kontaktflächen identifiziert und moduliert.

Noch steht die Virologie im Fokus, die Jahrestagung der Gesellschaft für Virologie im März 2023 in Ulm besuchten mehr als 800 Teilnehmende. Es gilt aber, für diese begeisterten jungen Wissenschaftlerinnen und Wissenschaftler Perspektiven zu schaffen, die diese wichtigen Forschungsarbeiten auch für die Zukunft sicherstellen. Hierfür sind auch gesetzliche Grundlagen notwendig, die gleichzeitig Flexibilität garantieren UND ein angemessenes Maß an Sicherheit bieten. Sind wir damit dann besser auf die nächste Pandemie vorbereitet? Die Probe aufs Exempel wird bestimmt kommen. ■

Funding note

Open Access funding enabled and organized by Projekt DEAL.

Prof. Dr. Dr. h.c. mult. Thomas C. Mettenleiter, Präsident des Friedrich-Loeffler-Instituts, Bundesforschungsinstitut für Tiergesundheit, Greifswald-Insel Riems


Literatur

[1] Sutle Curtis A Viruses in the sea Nature 2005 437 356 361 10.1038/nature04160 16163346
